# Engaging in Rather than Disengaging from Stress: Effective Coping and Perceived Control

**DOI:** 10.3389/fpsyg.2016.01415

**Published:** 2016-09-21

**Authors:** Maria T. M. Dijkstra, Astrid C. Homan

**Affiliations:** ^1^Organization Sciences, VU University AmsterdamAmsterdam, Netherlands; ^2^Work and Organizational Psychology, University of AmsterdamAmsterdam, Netherlands

**Keywords:** coping, perceived sense of control, stress, psychological well-being, (dis)engagement

## Abstract

Being able to cope effectively with stress can help people to avoid negative consequences for their psychological well-being. The purpose of this study was to find out why some coping strategies are effective in reducing the negative effect of stressors on well-being and some are not. We argue that the degree to which such coping strategies engage or disengage people from stressful incidents is related to their perceived control of the situation that, in turn, is positively associated with their psychological well-being. We thus propose that the relationship between coping and psychological well-being is mediated by the extent of perceived sense of control. We collected cross-sectional data from a large heterogeneous sample (*N* = 543) in the Netherlands. We assessed seven different coping strategies, perceived control, and psychological well-being. Our results indeed revealed that strategies reflecting more engaged coping such as active confronting and reassuring thoughts, were associated with more sense of control and therefore to psychological well-being. In contrast, strategies reflecting disengagement coping, such as passive reaction pattern, palliative reaction, and avoidance, were associated with less perceived control, which in turn was negatively associated with psychological well-being. Results regarding the coping strategies expressing emotions and seeking social support were less straightforward, with the former being negatively associated with perceived control and psychological well-being, even though this strategy has stress engaging elements, and the latter only showing a positive indirect effect on psychological well-being via perceived control, but no positive main effect on well-being. These findings are discussed from the perspective of stress being an environment-perception-response process.

## Introduction

One of the most well-documented and supported effects in stress research includes the finding that stressful life events significantly increase health risks in terms of cardiovascular disease (Hemingway and Marmot, 1999; Steptoe and Kivimäki, 2012; Buckley and Shivkumar, 2016), cancer (Antoni et al., 2006; Bower et al., 2014), and depression (Slavich and Irwin, 2014). Additionally, stress at the workplace is related to turnover (Bridger et al., 2013) and loss of productivity (for a review see Wolever et al., 2012). The associated economic costs of stress-induced outcomes as estimated by the International Labor Organization (ILO) amounted to EUR 9.2 billion in the EU, £1.1–1.2 billion in the U.K and USD 6.6 billion in the US (Mino et al., 2006). More recently, according to the statistical data released by the UK Health and Safety Executive (2013/2014), work-related stress, depression, or anxiety accounted for 39% (487,000 cases) of all work-related illnesses, with 11.3 million workdays being lost.

It is therefore crucial to understand the stress process in order to avoid or at least reduce its harmful physical, psychological, and economic consequence. Conducting such research is even more relevant in light of the global and severe stressful event that constitutes the financial and economic crisis, which started in the US in 2007–2008 and has spread around world, continuing in many countries today (Acharya et al., 2009; Goodman and Mance, 2011; Giorgi et al., 2014; Mucci et al., 2016).

Importantly, what specific outcomes the stress process will generate, and how severe its consequences are, largely depends on the way people handle potentially threatening events (e.g., unemployment, increased workload), referred to as stressors. Coping with stressors, therefore, became a key subject within stress literature (e.g., Carver et al., 1989; Aspinwall and Taylor, 1997; Skinner et al., 2003; Inzlicht and Kang, 2010).

Coping refers to as a person's efforts to manage demands that are appraised as taxing or exceeding their resources (Lazarus and Folkman, 1984). Coping can explain how stressors affect people and when and why people experience more or less detrimental consequences in terms of health and well-being (Skinner et al., 2003). At present, there is theoretical as well as empirical evidence that coping matters and that some coping strategies work better than others (e.g., Skinner et al., 2003; Britt et al., 2016). It is however unclear *why* certain coping strategies have more beneficial effects than others. In this study we wish to provide an answer to that question.

### Coping with stressful life events

Stress is most generally agreed upon as to be an environment-perception-response process (e.g., Spector and Jex, 1998; Pindek and Spector, 2016). As such it aligns with the transactional stress theory (Lazarus, 1991; Perrewe and Zellars, 1999) holding that environmental stressors (e.g., organizational constraints, economic crises) are experienced as threats to well-being that consequently lead to strains.

Within this theoretical framework, research on stress is concerned with the (inadequate) adaptation of individuals to environmental demands that are appraised as taxing or exceeding a person's resources (Lazarus and Folkman, 1984). In spite of their high potential for harming the health and well-being of people (Hemingway and Marmot, 1999; Antoni et al., 2006; Garcia-Bueno et al., 2008), stressors do not automatically induce such negative effects. What consequences stressors have largely depend on the specific behavior that people engage in or on the technique(s) they use to handle the particular threatening condition or event in order to counteract its effects on their well-being (Moos, 1990; Le Fevre et al., 2006; Dijkstra et al., 2009; Bridger et al., 2013). In other words, it is not primarily the stressor; it is how people cope with the stressor that will determine the consequences for health and well-being.

Aspinwall and Taylor (1997) define coping as “*activities* undertaken to *master, tolerate, reduce, or minimize* environmental or intra-psychic demands perceived to represent potential threats, existing harm, or losses” (p. 417; see also Lazarus and Folkman, 1984; Folkman and Lazarus, 1985; Latack, 1986). Previous research has shown that different coping strategies differ in their success in influencing stress-related outcomes. For instance, Koeske et al. (1993) showed in a study among case managers who were working with clients with mental illness, that active coping styles served as work stress buffers while the use of avoiding strategies resulted in higher general levels of negative consequences three months later. Similarly, research among bank employees revealed that initiating direct action in response to a stressor positively predicted job satisfaction and negatively predicted psychological distress (Fortes-Ferreira et al., 2006). In addition, a study on depression among medical students revealed that students who were likely to use avoidant coping strategies, and unlikely to engage in active strategies tended to have higher stress levels (Chu-Lien Chao, 2011). Furthermore, in a study among patients with type 2 diabetes mellitus, Shah et al. (2012) showed that the group of patients that used more avoidance and passive resignation coping reported more depressive symptoms. Finally, Britt et al. (2016) examined the effectiveness of coping strategies in military basic training, a work environment characterized by the presence of unavoidable demands and low autonomy. Results showed that more use of the “active coping” strategy was related to a reduction in mental health symptoms, whereas the greater use of the coping strategy “denial/self-criticism” was associated with an increase in mental health symptoms.

These previous results strongly suggest that coping strategies reflecting direct “action,” thereby facing the stressor and/or its related emotions, are more successful in preventing negative consequences of stress, than more avoidant types of coping, which imply diverting from the stressor and/or its related emotions. The latter types of coping have shown to “typically work against people” (Carver and Scheier, 1994, p. 184).

Despite the numerous categories of coping (for a review see Skinner et al., 2003), we follow Carver and Connor-Smith (2010) in their conclusion that “The distinction that appears to have greatest importance is engagement vs. disengagement” (p. 687). Their “engagement” category of coping reflects coping strategies in which “a person takes charge in tone” (Latack, 1986, p. 378), by facing the stressor and/or its related emotions. The “disengagement” category of coping involves strategies that are aimed at diverting from the stressor and/or its related emotions. Research has shown that people who use disengagement coping generally are not able to deal with the stressor and as a result are more likely to experience the negative consequences of the stressor compared to people who engage in more active coping strategies (e.g., Fortes-Ferreira et al., 2006; Chu-Lien Chao, 2011). We propose that these effects are driven by the degree to which these coping strategies provide the person with a sense of being in control of the stressful situation.

During the past decades, research has demonstrated that people's ability to gain and maintain a sense of control is an important personal resource (Turner, 1988), is associated with their well-being being (Mirowsky and Ross, 1990; Thoits, 1995; Turner and Lloyd, 1999; Chipperfield et al., 2004; Dijkstra et al., 2011) and is essential for their evolutionary survival (Shapiro et al., 1996). In a recent study, Vander Elst et al. (2016) showed that personal control mediated the relationship between job insecurity (serving as the stressor) and physical and mental health complaints. Based on these studies, we argue that perceived control might be the driving ingredient that explains why engagement coping strategies have more positive effects for psychological well-being than disengagement strategies. People who actively deal with the situation at hand, are more likely to experience that they are in charge of the situation and are able to change something about it, whereas those that avoid or ignore stressful events experience a lack of possibilities to confront the stressor (Latack, 1986; Dijkstra et al., 2009).

Below, we present a cross-sectional study run in The Netherlands, in which we examined the role of perceived control as an explaining mechanism underlying successful and unsuccessful coping efforts. To assess the coping strategies respondents employed, we used the Utrecht Coping List (UCL; Schreurs et al., 1993) because this is a validated and originally Dutch scale. The scale contains seven subscales, which, as we propose below, could be categorized into either engagement or disengagement coping.

The first coping style, active confronting, refers to directly and consciously facing the stressor. Handling the stressor in this “hands on” way embodies an engagement coping strategy. Second, seeking social support means actively asking others to help you. By involving others to eliminate or at least diminish the stressful event, one employs an engagement coping strategy. Third, reassuring thoughts signifies putting things in perspective and looking for ways to acknowledge that “it's not the end of the world.” By taking such a course of action one exercises an engagement coping strategy. Fourth, expressing emotions refers to explicitly venting feelings and therefore represents an engagement coping strategy. Fifth, passive reaction pattern is “wallowing” in negativity and not really addressing the stressor, but instead in a helpless kind of way, letting your self be submerged by the stressful event. Responding like this reflects a disengagement coping strategy. Sixth, palliative reaction denotes a disassociating from the stressor by letting the stressful event numb you. As such, this coping strategy represents a disengagement coping strategy. Finally, avoiding is taking a lethargic stance toward the stressor, which symbolizes a disengagement coping strategy.

**Figure 1:**
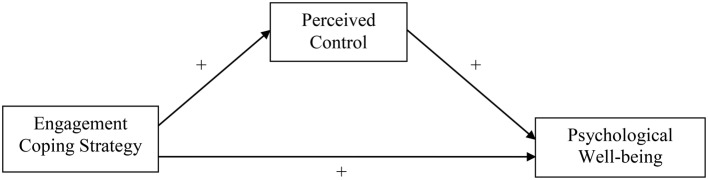
**Conceptual model capturing the proposed relationships between engagement coping strategy, perceived control, and psychological well-being**.

**Figure 2:**
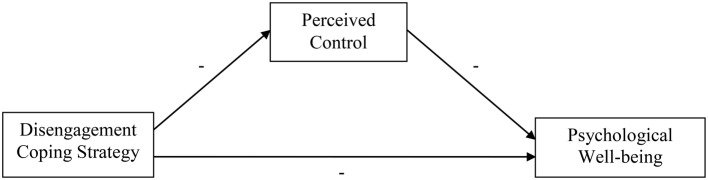
**Conceptual model capturing the proposed relationships between disengagement coping strategy, perceived control, and psychological well-being**.

In sum, we predict that the relationship between coping with stressors and reduced psychological well-being strongly depends on the amount of perceived control that is related to the particular coping strategy. More specifically, we predict that strategies reflecting more engaged coping (i.e., active confronting, seeking social support, reassuring thoughts, and expressing emotions) will be related to more perceived control and in turn to better psychological well-being. Strategies reflecting more disengaged coping (i.e., passive reaction pattern, palliative reaction, and avoidance) on the other hand will be related to less perceived control and therefore to less psychological well-being (see Figures 1, 2).

## Materials and methods

### Sample and procedure

Data were collected in the context of a broader research question concerning coping, well-being, and cultural differences between Hindustan and other Dutch inhabitants of The Netherlands (Ramlal, 2007). The study was carried out in accordance with the recommendations of the ethics regulations of Leiden University. At the time of the study (in 2006), Leiden University did not have an ethics committee for this type of unobtrusive research projects, which implied that the study did not receive an IRB number. All respondents received an information brochure from the research team, inviting them to participate in the study emphasizing the importance of participating as well as its voluntary and anonymous character. Sample participants filled in the questionnaires either by paper and pencil or online. Some participants were approached in public spaces, whereas others were approached online.

Out of 575 questionnaires 543 were returned (response rate: 94.44%). Of all respondents 279 (51.4%) were male and the mean age was 35.20 years (*SD* = 12.65; range 15–75). Parents provided active consent for the 11 minors in our sample. Analysing the data excluding the eleven minors did not change the pattern or conclusion of the results. Respondents were categorized as Caucasian-Dutch (37.3%, *n* = 202) or non-Caucasian-Dutch ethnicity. The educational level of the employees in our sample ranged from elementary school (1.8%, *n* = 10) through mid-level (40.7%, *n* = 220) and higher level (38.4%, *n* = 208) vocational education, university (16.5%, *n* = 89) to post-academic education (2.6 %, *n* = 14). Two participants had missing values for gender, ethnicity, and educational level. Three participants did not report their age.

### Measures

#### Psychological well-being

We used the five-item version of the Mental Health Inventory (MHI-5) developed by Veit and Ware (1983) which has proven to be equally good as the MHI-18 (Berwick et al., 1991). Participants were asked, “How much of the time, during the last month, have you…” and a sample item was,“…been a happy person.” Response categories ranged from 1 (all of the time) to 6 (none of the time). Cronbach's alpha for the scale was 0.84. Higher scores on the scale indicate more favorable health.

#### Coping strategy

To measure coping strategy, the Utrecht Coping List (UCL; Schreurs et al., 1993) was used. This is a validated questionnaire (47 items) that quantifies dispositional coping characteristics for problems and unpleasant events in daily life. In contrast to state measures, trait measures like the UCL allow for greater comparison of coping styles across different samples and situations.

Participants responded to the question “how often do the following behaviors apply to you?” Each item had four response choices, ranging from 1 (never) to 4 (very often). Sub-scores were used to describe individual tendency to seven coping strategies: (a) Active confronting involved eight items (Cronbach's alpha was 0.83) and a sample item was “In order to solve the problem I consciously addressed the matter.” (b) Seeking social support involved five items (α = 0.80), and a sample item was: “I asked someone for help.” (c) Reassuring thoughts was measured with seven items (α = 0.70). A sample item was “I realized more serious things can happen.” (d) Expressing emotions involved five items (α = 0.68; e.g., “I showed my anger toward the person that was responsible for the problem”). (e) Passive reaction pattern was assessed with seven items (α = 0.73), and a sample item was “I was overpowered by the problem.” (f) Palliative reaction involved nine items (α = 0.74) and a sample item was “I tried to think of things that were not related to the problem.” (g) Finally, avoidance was measured with six items (α = 0.67) and a sample item was “I let things happen.” A higher score indicated an increased tendency toward that particular coping strategy.

#### Perceived sense of control

We used 4 items developed by Cohen et al. (1983) that rated the frequency of their feelings of control related to events and situations that occurred in the last month (α = 0.78). A sample item was, “How often have you felt that you were not in control of important things in your life?” Each item was rated on a five point Likert-type scale ranging from 1 (never) to 5 (very often). Higher scores indicated less perceived control.

#### Control variables

Past work on stress at work revealed gender differences in coping with stressors (e.g., Ptacek et al., 1994; Almeida and Kessler, 1998; Matud, 2004). Furthermore, there are consistent gender differences in anxiety and depression (e.g., Barnett et al., 1987; Nolen-Hoeksema, 1987). We thus included gender as a control variable (dummy-coded: 0 = male, 1 = female) Like gender, age seemed a relevant control variable as well. Not only does age correlate with well-being (Siu et al., 2001), it is also associated with control beliefs and locus of control (Lachman and Weaver, 1998). We also controlled for education level because it has also been associated with well-being (Bryant and Veroff, 1984). Educational background was coded on a 5-point scale (higher numbers represent higher educational level). Finally, as the study was originally developed to examine differences between Hindustan and other Dutch inhabitants of The Netherlands, we controlled for participant cultural background (dummy-coded: 0 = Dutch, 1 = Hindustani).

## Results

### Descriptive statistics

Means, standard deviations, and zero-order correlations among study variables can be found in Table 1. The control variables cultural background, sex, age, and educational background showed some significant, but no consistent, relationships with the key variables in our study. In order to account for these relationships, we controlled for these variables when testing our predictions.

**Table 1:** **Means, standard deviations, and correlations (***N*** = between 537 and 543)**.

	**Mean**	***SD***	**1**	**2**	**3**	**4**	**5**	**6**	**7**	**8**	**9**	**10**	**11**	**12**	**13**	**14**
1. Cultural background	0.63	0.48														
2. Sex	0.48	0.50	0.01													
3. Age	35.20	12.65	−0.07	−0.11^*^												
4. Educational background	2.77	0.84	−0.17^**^	0.02	−0.16^**^											
5. Active confronting	2.63	0.56	0.02	−0.06	0.14^*^	0.09^*^										
6. Seeking social support	2.12	0.65	−0.08	0.32^**^	−0.24^**^	0.06	0.23^**^									
7. Reassuring thoughts	2.41	0.53	0.15^**^	0.13^*^	0.07	0.03	0.61^**^	0.29^**^								
8. Passive reaction pattern	1.90	0.52	0.21^**^	0.12^*^	−0.21^**^	−0.06	−0.16^**^	0.13^**^	0.11^**^							
9. Palliative reaction	2.10	0.48	0.08	0.14^*^	−16^**^	0.05	0.16^**^	0.34^**^	0.36^**^	0.48^**^						
10. Avoidance	1.93	0.52	0.16^*^	0.03	−0.21^**^	−0.09	−0.11^*^	0.14^**^	0.17^**^	0.61^**^	0.48^**^					
11. Expressing emotions	2.18	0.54	−0.05	0.29^**^	−0.14^**^	0.06	0.23^**^	0.59^**^	0.30^**^	0.22^**^	0.40^**^	0.22^**^				
12. Engagement coping	2.39	0.44	0.03	0.18^**^	−0.03	0.08	0.78^**^	0.70^**^	0.80^**^	0.04	0.38^**^	0.09^*^	0.51^**^			
13. Disengagement coping	1.98	0.42	0.18^*^	0.11^**^	−0.24^**^	−0.05	0.05	0.24^**^	0.25^**^	0.85^**^	0.78^**^	0.85^**^	0.33^**^	0.20^**^		
14. Perceived control	3.43	0.82	−0.06	−0.21^**^	0.21^**^	0.03	0.38^**^	−0.16^**^	0.11^*^	−0.63^**^	−0.35^**^	−0.41^**^	−0.25^**^	0.12^**^	−0.55^**^	
15. Psychological well-being	4.15	0.90	−0.15^**^	−0.11^**^	0.13^**^	0.03	0.33^**^	−0.00	0.11^*^	−0.63^**^	−0.27^**^	−0.35^**^	−0.11^*^	0.18^**^	−0.46^**^	0.68^**^

*Cultural background (0 = Dutch, 1 = Hindustani) and sex (0 = male; 1 = female) were dummy-coded. Educational background was coded based on a 5-point scale (higher numbers represent higher educational level). Engagement coping combines active confronting, seeking social support, and reassuring thoughts into one composite scale. Disengagement coping combines passive reaction pattern, palliative reaction, and avoidance into one composite scale*.

* p < 0.05;

** *p < 0.01*.

### Data analyses

We regressed psychological well-being and perceived control on the seven coping strategy variables. We applied a Bonferroni correction by adjusting the test for significant effects relative to the number of repeated analyses. More specifically, we divided our *p*-value of 0.05 by seven, because we ran a separate analysis for every different coping style (adjusted cut-off *p*-value = 0.007). In the first step we entered the control variables, and in the second step we entered the main effect of the coping strategy of interest. Finally, we tested mediation by perceived control in the relationship between coping and psychological well-being following the mediation analysis approach of Baron and Kenny (1986). Results are shown in Tables 2, 3. Additionally, we employed the PROCESS procedure for SPSS (Hayes, 2013; Model 4) calculating bias-corrected confidence intervals (CI) to examine the indirect effect based on 10,000 bootstrap samples (Preacher and Hayes, 2004; Hayes, 2013). If the CI excludes zero, there is evidence for a significant indirect effect.

**Table 2:** **Results of hierarchical regression analyses examining the role of separate coping strategies on perceived control (***N*** = 536)**.

	**Coping strategy**						
	**Active confronting**	**Seeking social Support**	**Reassuring thoughts**	**Expressing emotions**	**Passive reaction pattern**	**Palliative reaction**	**Avoidance**
**MODEL, R SQUARE CHANGE**							
1	0.09^*^	0.09^*^	0.09^*^	0.09^*^	0.09^*^	0.09^*^	0.09^*^
2	0.12^*^	0.00	0.02^*^	0.03^*^	0.33^*^	0.09^*^	0.13^*^
**STEP 1 (CONTROLS)**							
Cultural background	−0.05	−0.05	−0.05	−0.05	−0.05	−0.05	−0.05
Sex	−0.19^*^	−0.19^*^	−0.19^*^	−0.19^*^	−0.19^*^	−0.19^*^	−0.19^*^
Age	0.19^*^	0.19^*^	0.19^*^	0.19^*^	0.19^*^	0.19^*^	0.19^*^
Education	0.04	0.04	0.04	0.04	0.04	0.04	0.04
**STEP 2 (MAIN EFFECT)**							
Coping strategy	0.36^*^	0.07	0.13^*^	−0.18^*^	−0.60^*^	−0.30^*^	−0.38^*^
*R*^2^	0.21^*^	0.09^*^	0.10^*^	0.12^*^	0.42^*^	0.17^*^	0.22^*^

*Standardized regression weights are reported (β). Step 1 results are the same for all coping strategies. Cultural background (0 = Dutch-Caucasian, 1 = Other) and sex (0 = male; 1 = female) were dummy-coded. Educational background was coded based on a 5-point scale (higher numbers represent higher educational level). We used a Bonferroni corrected p-value for significance testing*.

* *p < 0.007*.

**Table 3:** **Results of hierarchical regression analyses examining the role of separate coping strategies on psychological well-being and mediation by perceived control (***N*** = 536)**.

	**Coping strategy**						
	**Active confronting**	**Seeking social support**	**Reassuring thoughts**	**Expressing emotions**	**Passive reaction pattern**	**Palliative reaction**	**Avoidance**
**MODEL, R SQUARE CHANGE**							
1	0.05^*^	0.05^*^	0.05^*^	0.05^*^	0.05^*^	0.05^*^	0.05^*^
2	0.10^*^	0.00	0.02^*^	0.01	0.35^*^	0.06^*^	0.10^*^
3	0.43^*^	0.43^*^	0.42^*^	0.47^*^	0.48^*^	0.42^*^	0.43^*^
**STEP 1 (CONTROLS)**							
Cultural background	−0.14^*^	−0.14^*^	−0.14^*^	−0.14^*^	−0.14^*^	−0.14^*^	−0.14^*^
Sex	−0.10	−0.10	−0.10	−0.10	−0.10	−0.10	−0.10
Age	0.11	0.11	0.11	0.11	0.11	0.11	0.11
Education	0.03	0.03	0.03	0.03	0.03	0.03	0.03
**STEP 2 (MAIN EFFECT)**							
Coping strategy	0.32^*^	0.05	0.14^*^	−0.08	−0.62^*^	−0.24^*^	−0.33^*^
**STEP 3 (MEDIATION)**							
Coping strategy	0.09^*^	0.10^*^	0.05	0.05	−0.32^*^	−0.04	−0.08
Perceived control	0.64^*^	0.69^*^	0.67^*^	0.68^*^	0.48^*^	0.67^*^	0.65^*^
*R*^2^ *(step 1 and 2)*	0.14^*^	0.05^*^	0.07^*^	0.06^*^	0.39^*^	0.10^*^	0.15^*^
*R*^2^ *(step 3)*	0.48^*^	0.48^*^	0.47^*^	0.42^*^	0.53^*^	0.47^*^	0.48^*^

*Standardized regression weights are reported (β). Step 1 results are the same for all coping strategies. Cultural background (0 = Dutch-Caucasian, 1 = Other) and sex (0 = male; 1 = female) were dummy-coded. Educational background was coded based on a 5-point scale (higher numbers represent higher educational level). We used a Bonferroni corrected p-value for significance testing*.

* *p < 0.007*.

### Engagement coping

We argued that active confronting, seeking social support, reassuring thoughts, and expressing emotions would be more engaged coping styles. We proposed that more engaged coping styles should be associated with higher perceived control, which in turn should be associated with higher psychological well-being.

#### Active confronting

People who scored higher on active confronting experienced a higher sense of control and higher psychological well-being. When including both active confronting and perceived sense of control in the regression analysis, we found that the strength of the relationship between active confronting and psychological well-being was reduced, while the effect of perceived sense of control was significant, supporting partial mediation. A Sobel test indicated that the mediation was significant (*z* = 8.09, *p* < 0.001). Similarly, the bootstrap approach supported an indirect effect between active confronting and psychological well-being via perceived sense of control (point estimate = 0.37, *SE* = 0.05, 99% bias corrected CI [0.25, 0.50]). Indeed, this suggests that people who use more active confronting reported higher well-being due to a higher perceived sense of control.

#### Seeking social support

Opposite to our reasoning, seeking social support was not significantly associated with perceived control and psychological well-being. We thus found no support for seeking social support as a potentially effective coping strategy.

#### Reassuring thoughts

People who scored higher on reassuring thoughts experienced a higher sense of control and higher psychological well-being. When including both reassuring thoughts and perceived sense of control in the regression analysis, we found that the relationship between reassuring thoughts and psychological well-being was no longer significant, while the effect of perceived sense of control was significant, supporting full mediation (Sobel test: *z* = 8.24, *p* < 0.001). Similarly, the bootstrap approach showed that the indirect effect was significant (point estimate = 0.15, *SE* = 0.05, 99% bias corrected CI [0.02, 0.28]). These results imply that people who use more reassuring thoughts reported higher well-being due to a higher perceived sense of control.

#### Expressing emotions

Expressing emotions was not significantly associated with psychological well-being, and negatively associated with perceived control. We thus found no support for expressing emotions as a potentially effective coping strategy. We did test for a potential indirect effect using the bootstrap approach (point estimate = −0.21, *SE* = 0.06, 99% bias corrected CI [−0.36, −0.07]). Even though expressing emotions was not directly associated with psychological well-being, this analysis indicates that there was an indirect effect via perceived control. However, in contrast to our theoretical reasoning, this relationship was negative rather than positive.

### Disengagement coping

We argued that passive reaction pattern, palliative reaction, and avoidance would be more disengaged coping styles. For these disengaged coping styles, we argued these are associated with lower perceived control, which in turn should be associated with lower psychological well-being.

#### Passive reaction pattern

People who scored higher on passive reaction pattern experienced a lower sense of control and lower psychological well-being. When including both passive reaction pattern and perceived sense of control in the regression analysis, we found that the strength of the relationship between passive reaction pattern and psychological well-being was reduced, while the effect of perceived sense of control was significant, supporting partial mediation. A Sobel test indicated that this mediation was significant (*z* = 7.31, *p* < 0.001). Similarly, the bootstrap approach supported an indirect effect between relationship between passive reaction pattern and psychological well-being via perceived sense of control (point estimate = −0.51, *SE* = 0.06, 99% bias corrected CI [−0.69, −0.36]). Indeed, it seems to be the case that people who use more passive reaction pattern reported lower well-being due to a reduced perceived sense of control.

#### Palliative reaction

Palliative reaction showed a negative relationship with both perceived control and psychological well-being. When entering both palliative reaction and perceived control in the analysis, the original significant association between palliative coping and psychological well-being became non-significant, while the association between perceived control and psychological well-being was significant. The Sobel test was significant (*z* = 8.17, *p* < 0.001). The bootstrap approach also supported a significant indirect effect (point estimate = −0.38, *SE* = 0.06, 99% bias corrected CI [−0.54, −0.23]).

#### Avoidance

People who scored higher on avoidance experienced a lower sense of control and lower psychological well-being. When including both avoidance and perceived sense of control in the regression analysis, we found support for full mediation (*z* = 8.09, *p* < 0.001). Similarly, the bootstrap approach showed that the indirect effect was significant (point estimate = −0.43, *SE* = 0.06, 99% bias corrected CI [−0.57, −0.29]). These results suggest that people who use more avoidance reported lower well-being due to a lower perceived sense of control.

### Exploratory factor analysis

Above, we reported the analyses for each coping strategy separately. However, we categorized different coping strategies in engagement and disengagement coping. To probe whether our data support this distinction in coping strategies, we subjected the 47 coping items of the the Utrecht Coping List (UCL; Schreurs et al., 1993) to an exploratory factor analysis with varimax rotation requesting two factors. This first factor analysis showed that the expressing emotions items did not clearly distinguish between the two factors. We therefore omitted these items from the analyses below, and subjected the remaining 42 items to another exploratory factor analysis requesting two factors. The results indicated that the items assessing active confronting, seeking social support, and reassuring thoughts loaded high on the “engagement” factor (20 items; Eigenvalue = 6.29; variance explained = 15.00%), whereas the items measuring passive reaction pattern, palliative reaction, and avoidance loaded high on the “disengagement” factor (22 items; Eigenvalue = 6.06; variance explained = 14.42%). Consequently, we computed an engagement (*M* = 2.39, *SD* = 0.44; α = 0.85) and a disengagement (*M* = 1.98, *SD* = 0.42; α = 0.85) coping strategy variable. In an exploratory fashion, we re-ran the analyses for these two composite coping variables (see Table 4).

**Table 4:** **Results of hierarchical regression analyses examining the role of the composite coping strategy variables on perceived control and psychological well-being and the mediating role of perceived control (***N*** = 536)**.

	**Perceived control**		**Psychological well-being**	
	**Coping strategy**		**Coping strategy**	
	**Engagement**	**Disengagement**	**Engagement**	**Disengagement**
**MODEL, R SQUARE CHANGE**				
1	0.09^***^	0.09^***^	0.05^***^	0.05^***^
2	0.03^***^	0.26^***^	0.04^***^	0.22^***^
2a			0.43^***^	0.44^***^
**STEP 1 (CONTROLS)**				
Cultural background	−0.05		−0.14^*^	
Sex	−0.19^***^		−0.10^*^	
Age	0.19^***^		0.11^*^	
Education	0.04		0.03	
**STEP 2 (MAIN EFFECT)**				
Coping strategy	0.18^***^	−0.53^***^	0.21^***^	−0.49^***^
*R*^2^	0.12^***^	0.34^***^	0.09^***^	0.27^***^
**STEP 2A (MEDIATION)**				
Coping strategy			0.10^**^	−0.18^***^
Perceived control			0.66^***^	0.59^***^
*R*^2^			0.48^***^	0.49^***^

*Standardized regression weights are reported (β). Step 1 results are the same for both coping strategies. Cultural background (0 = Dutch-Caucasian, 1 = Other) and sex (0 = male; 1 = female) were dummy-coded. Educational background was coded based on a 5-point scale (higher numbers represent higher educational level)*.

* p < 0.05;

** p < 0.01;

*** *p < 0.001*.

#### Engagement coping composite variable

People who scored higher on engagement coping experienced a higher sense of control and higher psychological well-being. When including both engagement coping and perceived sense of control in the regression analysis, we found that the strength of the relationship between engagement coping and psychological well-being was reduced, while the effect of perceived sense of control remained significant, supporting partial mediation (Sobel test: *z* = 4.09, *p* < 0.001). The bootstrap approach supported a significant indirect effect (point estimate = 0.24, *SE* = 0.06, 99% bias corrected CI [0.09, 0.40]). Again, it seems to be the case that people who use more engagement coping strategies reported higher well-being due to a higher perceived sense of control.

#### Disengagement coping composite variable

Disengagement coping was negatively related to perceived sense of control and psychological well-being. When including both disengagement coping and perceived sense of control in the analysis, the strength of the relationship between disengagement coping and psychological well-being was reduced, while the effect of perceived sense of control stayed significant, indicating partial mediation (Sobel's *z* = 10.46, *p* < 0.001). Again, the bootstrap approach supported the mediating role of perceived control in the relationship between disengagement coping and psychological well-being (point estimate = −0.67, *SE* = 0.08, 99% bias corrected CI [−0.88, −0.50]). These findings lend support for our prediction that a disengaged way of handling stressors is related to lower well-being because it is associated with less perceived control.

### Testing alternative models with different directionality

Given the cross-sectional nature of our study, we also examined two alternative models in an exploratory fashion. In the first alternative indirect effect model, we examined perceived control as independent variable, coping strategies as mediator, and psychological well-being as dependent variable. The PROCESS results show weaker support for this model than for our theoretical model (see Table 5). These weaker effects were also found for the two composite coping strategy variables (for engagement strategies: point estimate = 0.02, *SE* = 0.01, 99% bias corrected CI [0.00, 0.05]; for disengagement strategies: point estimate = 0.10, *SE* = 0.03, 99% bias corrected CI [0.04, 0.19].

**Table 5:** **Indirect effects with 99% bias-corrected CI's pertaining to the proposed theoretical model and two alternative models**.

	**Theoretical model: Coping 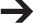 Perceived control 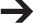 Psychological well-being**	**Alternative model 1: Perceived control 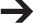 Coping 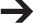 Psychological well-being**	**Alternative model 2: Psychological Well-being 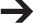 Perceived control 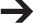 Coping**
**POINT ESTIMATES**			
Active confronting	**0.37 (0.05)**	**0.04 (0.02)**	**0.11 (0.02)**
Seeking social support	−0.06 (0.05)	−0.01 (0.01)	−0.08 (0.03)
Reassuring thoughts	**0.15 (0.05)**	0.01 (0.01)	0.03 (0.02)
Expressing emotions	−**0.21 (0.06)**	−0.01 (0.01)	−**0.09 (0.03)**
Passive reaction pattern	−**0.51 (0.06)**	**0.22 (0.03)**	−**0.13 (0.02)**
Palliative reaction	−**0.38 (0.06)**	0.01 (0.01)	−**0.09 (0.02)**
Avoidance	−**0.43 (0.06)**	**0.03 (0.02)**	−**0.11 (0.02)**
**99% CI's**			
Active confronting	**0.25, 0.50**	**0.00, 0.09**	**0.05, 0.17**
Seeking social support	−0.18, 0.05	−0.03, 0.00	−0.15, 0.00
Reassuring thoughts	**0.02, 0.28**	−0.01, 0.03	−0.03, 0.09
Expressing emotions	−**0.36**, −**0.07**	−0.04, 0.01	−**0.16**, −**0.02**
Passive reaction pattern	−**0.69**, −**0.36**	**0.13, 0.31**	−**0.19**, −**0.10**
Palliative reaction	−**0.54**, −**0.23**	−0.02, 0.05	−**0.15**, −**0.04**
Avoidance	−**0.57**, −**0.29**	**0.00, 0.09**	−**0.17**, −**0.06**

*Standard errors are reported in parentheses. Significant conditional indirect effect estimates and CI's are typed bold. In the second column, the specific coping strategy was tested as the independent variable. In the third column, the specific coping strategy was tested as the mediator. Finally, in the fourth column, the specific coping strategy was tested as dependent variable*.

In the second alternative model, we tested the complete reversed directional model (from psychological well-being to control to coping strategies). Again, these results are weaker than for our theoretical model (see Table 5). The results concerning the two composite coping strategy variables (for engagement strategies: point estimate = 0.02, *SE* = 0.02, 99% bias corrected CI [−0.03, 0.07]; for disengagement strategies: point estimate = −0.11, SE = 0.02, 99% bias corrected CI [−0.16, −0.07] also became weaker. These exploratory analyses seem to suggest stronger support for our theoretical model than for models in which the direction of the relationships between the variables is (partially) reversed.

## Discussion

The purpose of this study was to find out why some coping strategies are effective in reducing the negative effect of stressors on well-being and some are not. We proposed that the use of more engaged coping strategies, aimed at taking charge in tone, by facing the stressor and/or its related emotions, are related to more perceived control, which in turn is positively related to psychological well-being. We further proposed that when employing more disengaged coping, aimed at diverting from the stressor and/or its related emotions, perceived control was also lower, which in turn was related to deteriorated psychological well-being.

Our results showed that the coping strategies of passive reaction pattern, palliative reaction and avoidance were consistently and negatively related to perceived control and therefore to less well-being. The commonality between these three strategies is that their coping behavior is directed at turning away from the stressful event and/or the emotions that go along side with it. Such disengaged behaviors lead a person to experience a lack of control, and potentially a lack of possibilities to confront the stressor (Latack, 1986; Dijkstra et al., 2009).

Our results concerning active confronting and reassuring thoughts revealed a positive relationship with perceived control and through perceived control with more well-being. Seeking social support, however, was not related to either well-being or control and therefore could not be regarded as an effective coping strategy. Although seeking social support clearly implies activity and could therefore be considered to be an engagement coping strategy and directed toward the stressor and/or its emotions, the strategy obviously is an exception where it concerns its association with perceived control. Elaborating on this further it can be argued that the extent to which seeking social support will be related to perceived control is contingent on the success of the search. In other words is seeking social support resulting in actually getting social support? If not, for example when asked support when in fear of job loss is being denied, a lack of possibilities to confront the stressor might be experienced. Indeed, in light of the importance of social support for health and well-being (Tian et al., 2013; Emadpoor et al., 2016) such rejection might function as an additional stressor (Platt et al., 2013).

The coping strategy expressing emotions also was not related to well-being but there was a significant relationship with perceived control. This relationship was, however, in the opposite direction of what we expected. In line with this inconsistent finding, our exploratory factor analysis indicated that expressing emotions did not clearly map onto engagement (or disengagement) coping, but was negatively related to control, and indirectly negatively to well-being. When examining the items pertaining to this coping style (e.g., “shown your feelings,” “let off steam,” “shown your frustration,” “shown that you were angry with those responsible for the problem”), it becomes clear that these items might indeed load on both these factors, given that some items indicate a more engaged and activating handling of the stressor (anger toward those responsible) and others might be less engaged and diverge more from the stressor (letting off steam). Finally, expressing anger or frustration might be associated with a lack of perceived control, given that these emotions do not help to reappraise or handle the stressor, and as such might be associated with diminished well-being (Leonard and Alison, 1999).

Whereas past research has clearly supported control as being relevant for well-being (Mirowsky and Ross, 1990; Thoits, 1995; Turner and Lloyd, 1999; Chipperfield et al., 2004; Dijkstra et al., 2011; Vander Elst et al., 2016) and even survival (Shapiro et al., 1996), our data provide preliminary evidence of a relationship between ways of coping and perceived sense of control. The main contribution of this study therefore is that it suggests an important role of control in the stress process and that it deepens our understanding of the differential effectiveness of different coping strategies (Britt et al., 2016). Indeed, perceived sense of control might be identified as an important explaining variable in the relationship between coping and psychological well-being. Rather than merely activating or de-activating someone, the lack of engagement implied by disengagement strategies is correlated to feeling that the situation is outside of someone's control, which in turn is associated with negative consequences.

In line with the theoretical reasoning that the most insightful distinction in coping strategies is engagement vs. disengagement (Carver and Connor-Smith, 2010), an exploratory factor analysis illustrated that most of the coping styles nicely mapped onto these two categories. Conceptually it makes sense that active confronting, seeking social support, and reassuring thoughts loaded onto an engagement factor, given that these items include strategies in which a person takes charge in tone, by facing the stressor and/or its related emotions. Similarly, passive reaction pattern, palliative reaction, and avoidance involve strategies that are aimed at diverting from the stressor and/or its related emotions. However, given that expressing emotions did not satisfactorily fit in with one of the two factors, and that reassuring thoughts when analyzed separately showed no effects, our findings also suggest that when categorizing coping styles into broader categories, certain styles might involve a variety of strategies and behaviors. This points to the importance of theoretical development based on fundamental research, and a potential more fine-grained examination of these relationships in future research.

### Limitations and future research

We acknowledge that our data are of cross-sectional nature. This means that we cannot claim that the relationships really are in the direction we propose them to be. We are relatively confident, however, that our results will replicate in a more robust research design. This confidence is based on our examination of two different exploratory indirect effect models in which (a) coping strategies were the mediator, perceived control the independent variable, and psychological well-being as the outcome, and (b) psychological well-being influenced coping strategies via perceived control. Inspection of these models revealed (far) less strong indirect effects, some null-effects which were previously significant (e.g., for reassuring thoughts and palliative reaction), and confidence intervals much closer to zero. However, in order to develop stronger theoretical notions concerning the role of control in the stress process, a thorough examination of the different coping strategies as to their potential to elicit a sense of control is needed. Since its purpose of developing theory, such research would preferably be of experimental nature. Creating conditions in which different coping strategies are induced would allow for more causal explanations concerning the relationship between a particular coping strategy and perceived sense of control. These relationship could then be further examined conducting longitudinal research in order to test whether more engagement coping will indeed lead to more perceived control over time, which in turn might be positively related to psychological well-being.

Apart from the correlational nature of our study, a second limitation is the lack of information on the participant's locus of control. As locus of control has been discussed as a potential predictor of the use of certain coping strategies (e.g., Anderson, 1977), it would be interesting to control for personal locus of control in future research. However, locus of control is often seen as a relatively stable trait, which is difficult to influence. Our data seem to indicate that perceived control is influenced by coping strategies, and thus can vary depending on which coping style is employed. As the two composite coping styles are positively correlated, it seems unlikely that locus of control is an alternative explanation of our findings. Additionally, although our findings seem to indicate that influencing someone's sense of control might be a fruitful intervention for dealing with stress, future research might empirically test such an intervention.

Finally, in the current study, we did not investigate the context in which the coping strategy was employed. In line with the less consistent findings regarding seeking social support and expressing emotions, it might be the case that certain coping strategies are more or less needed or become more or less effective in certain contexts, for certain people, or in certain situations (Mucci et al., 2015; Reed, 2016). For instance, control might be more relevant to the degree that the stressful situation is more ambiguous, certain coping strategies might be more effective for more optimistic people, and one's standing in the organizational hierarchy might affect the opportunity to employ certain coping strategies. It would be interesting for future research to take such moderating factors into account.

## Conclusion

In sum, our data provide some preliminary insights into why different coping strategies can have negative or positive effects on psychological well-being. By increasing a sense of control, some coping strategies that are more engaging (i.e., active confronting and reassuring thoughts) are able to positively affect psychological well-being, whereas disengagement strategies (i.e., passive reaction pattern, palliative reaction, and avoidance) make people experience a lack of control and as such are likely to lower psychological well-being. Organizations could use these findings to actively coach and train their employees to seek effective control over stressful situations, and to teach them to employ engaged rather than disengaged coping styles.

## Author contributions

AH provided substantial contributions to the research conception and design. MD and AH analyzed and interpreted the data. MD wrote the paper, AH provided critical revisions of the paper. MD and AH both attended to the revision of the paper. MD and AH both approved of this version of the paper to be published. The authors thank Nishi Ramlal for collecting the data.

## Funding

This open access publication was made possible by a NWO Aspasia grant awarded to AH.

### Conflict of interest statement

The authors declare that the research was conducted in the absence of any commercial or financial relationships that could be construed as a potential conflict of interest.
